# The Role of Angiogenesis and Pro-Angiogenic Exosomes in Regenerative Dentistry

**DOI:** 10.3390/ijms20020406

**Published:** 2019-01-18

**Authors:** Alina-Andreea Zimta, Oana Baru, Mandra Badea, Smaranda Dana Buduru, Ioana Berindan-Neagoe

**Affiliations:** 1 MEDFUTURE-Research Center for Advanced Medicine, “Iuliu Hatieganu” University of Medicine and Pharmacy, 23 Marinescu Street, 400337 Cluj-Napoca, Romania; zimta.alina.andreea@gmail.com; 2 Department of Preventive Dentistry, Faculty of Dental Medicine, Iuliu Hatieganu University of Medicine and Pharmacy, 400083 Cluj-Napoca, Romania; oanabaru@gmail.com (O.B.); mindrabadea@yahoo.com (M.B.); 3 Prosthetics and Dental materials, Faculty of Dental Medicine, “Iuliu Hatieganu” University of Medicine and Pharmacy, Cluj-Napoca, 32 Clinicilor Street, 400006 Cluj-Napoca, Romania; 4 Stomestet Stomatology Clinic, Calea Manastur 68A Street, 400658 Cluj-Napoca, Romania; 5 Research Center for Functional Genomics, Biomedicine and Translational Medicine, “Iuliu Hatieganu” University of Medicine and Pharmacy, 23 Marinescu Street, 400337 Cluj-Napoca, Romania; 6 Department of Functional Genomics and Experimental Pathology, The Oncology Institute “Prof. Dr. Ion Chiricuta”, Republicii 34th street, 400015 Cluj-Napoca, Romania

**Keywords:** regenerative medicine, exosome, miRNA, angiogenesis, therapeutic delivery, stem cells

## Abstract

Dental surgeries can result in traumatic wounds that provoke major discomfort and have a high risk of infection. In recent years, density research has taken a keen interest in finding answers to this problem by looking at the latest results made in regenerative medicine and adapting them to the specificities of oral tissue. One of the undertaken directions is the study of angiogenesis as an integrative part of oral tissue regeneration. The stimulation of this process is intended to enhance the local availability of stem cells, oxygen levels, nutrient supply, and evacuation of toxic waste. For a successful stimulation of local angiogenesis, two major cellular components must be considered: the stem cells and the vascular endothelial cells. The exosomes are extracellular vesicles, which mediate the communication between two cell types. In regenerative dentistry, the analysis of exosome miRNA content taps into the extended communication between these cell types with the purpose of improving the regenerative potential of oral tissue. This review analyzes the stem cells available for the dentistry, the molecular cargo of their exosomes, and the possible implications these may have for a future therapeutic induction of angiogenesis in the oral wounds.

## 1. Introduction

Regenerative medicine intends to re-enforce the natural healing process of the human body with the purpose of maintaining the original tissue architecture [1] thus emulating as much as possible the high capacity of tissue and organ replacement, which characterizes simple life forms, such as the invertebrates *Planaria* sp. and *Hydra* sp. [2].

The adult stem cells represent the totality of cells that can regenerate, through differentiation, any type of tissue. These cells are first multiplied, then they are conditioned to differentiate into a specific cell type [3]. Through experimental manipulation, the differentiated mature cells can be also be reversed to a stem cell phenotype [4].

The advancements made in regenerative medicine have greatly influenced the field of dentistry. Regenerative dentistry uses the latest discoveries in stem cell research, material science, tissue engineering, and molecular biology in order to regenerate the tissues found in the oral cavity [5].

The formation of new blood vessels brings an efflux of nutrients and growth factors that will sustain the viability, proliferation and differentiation of the newly formed tissue structures. As follows, this process plays a fundamental role in a successful strategy of oral tissue regeneration [6,7].

The angiogenesis mechanism involves activation of the endothelial cells (EC) residing in the interior layer of a blood vessel, which results in the formation of a new blood vessel [8]. This process is required for the physiological wound healing [9], but it can also be a part of pathological processes, such as tumor development [10], stroke [11], and myocardial infarction [12].

**Figure 1:**
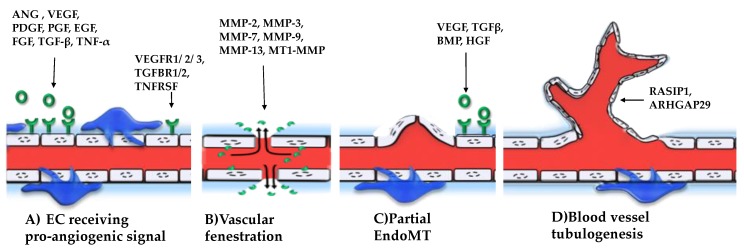
The angiogenic process has four main steps. (**A**) The endothelial cells (EC) found at the outer surface of a blood vessel, receive pro-angiogenic signals from the following factors: Angiogenin (ANG), Vascular Endothelial Growth Factor (VEGF), Platelet Derived Growth Factor (PDGF), Placental Growth Factor (PGF), Epidermal Growth Factor (EGF), Growth Factor Form Fibroblast (FGF), Transforming Growth Factor Beta 1 (TGF Β), and Tumor Necrosis Factor Alpha (TNF-α). The angiogenic growth factors have several corresponding receptors on the surface of EC, for instance VEGFR1/2/3, TGFΒR1/2, TNFRSF. After signal transduction in the EC, these cells start to produce metalloproteinases. (**B**) At the same time, the blood vessel pores have an increase size and because of this fenestration, the MMPs are able to escape from the blood vessel and degrade the basement membrane. (**C**) Then the ECs start to migrate, through a process called partial endothelial to mesenchymal transition (partial EndoMT) and proliferate at the place of fenestration, resulting in the budding of a new blood vessel. (**D**) As the new tube forms, there are multiple signals, such as RASIP1 and ARHGAP29, received from the environment that will give the 3D structure and organization of the newly formed network. By the end of this stage, the pericytes found at the exterior of the blood vessel responsible for blood vessel contraction are also beginning to populate the newly formed network.

The angiogenesis is composed of several stages. First, the surrounding cells release pro-angiogenic factors in the local microenvironment, which bind to their corresponding receptor found at the EC surface. This determines the ECs to proliferate and begin to secrete metalloproteinases (MMPs) that disrupt the basement membrane. The plasma proteins function as temporary scaffolds for cell migration [13]. The migration is mediated by several factors among which there are the Angiopoietin 1 (Ang1) and the αvβ5 integrin [14]. These stimulate the sprouting of a new blood vessel and establish the network architecture. Other cellular populations, such as the pericytes surround the newly formed blood vessel and finalize the angiogenic process [13]. A schematic representation of this process and the major factors involved in each step are illustrated in Figure 1.

During vascular fenestration and budding of a new blood vessel, the endothelial cells gain partial mesenchymal characteristics, through a process called partial endothelial to mesenchymal transition (partial EndoMT) meaning that the cytoskeleton organization is changed, the cells are more invasive, but they maintain the intracellular connection. During early in utero life, this process has major implications for the formation of new blood vessels [15,16]. In a mature organism, it is a key stage in the neovascularization of wound sites, which is why it has strong implications in regenerative medicine [17]. However, the overstimulation of this process can lead to fibrosis and cardiovascular diseases [18]. 

The angiogenesis is regulated by a number of molecular mediators. There are several growth factors: Vascular Endothelial Growth Factors (VEGF) [19], Platelet Derived Growth Factor (PDGF) [20], Placental Growth Factor (PGF) [21], Epidermal Growth Factor (EGF) [22], Fibroblast Growth Factor (FGF), Angiopoietin (ANG), Transforming Growth Factor-β (TGF β) and Tumor Necrosis Factor-alpha (TNF-α) [19]. The growth factors work in conjunction with a large amount of MMPs which degrade the extracellular matrix, meaning: MMP-2, MMP-3, MMP-7, MMP-9, MMP-13, and MT1-MMP [23]. 

The VEGF family of molecules bind to the heparan sulphate proteoglycans [24,25]. There are 5 members of the VEGF family: VEGFA, VEGFB, VEGFC, VEGFD, and PDGF. A different gene located on a different chromosome encodes each of the VEGF types. Aside from this heterogeneity, each member of the VEGF family can give rise to different variants due to the alternative spicing [26]. The VEGF increases the release of the Von Willenbrand factor involved in clot formation [27,28] and it determines the fenestration of a blood vessels [29]. In order to minimalize the side effects of VEGF induced therapeutic overexpression, the VEGF delivery through fibrin or collagen scaffold is used, because it determines the continuous release of VEGF thus minimizing side effects [30,31].

In the present review, we summarize the various tools available for the therapeutic stimulation of angiogenesis in regenerative dentistry, especially with the help of the stem cells and their exosomes. The exosomes are mediators of intercellular communication and can be used to maximize the local pro-angiogenic potential at the wound site. We also present a number of pro- and anti-angiogenic microRNAs, which modulate the behavior of endothelial cells and could be included in the design of exosomal delivery of pro-angiogenic factors with the purpose of enhancing the therapeutic regeneration of oral wounds.

## 2. Stem Cells Present in the Oral Cavity—Their Regenerative and Pro-Angiogenic Capacity

### 2.1. General Considerations

The stem cells are undifferentiated cells with great self-renewing capacity. They have different degrees of differentiation: totipotent, pluripotent, and multipotent. The totipotent stem cells are found only in the early stages of embryonic development. The pluripotent stem cells can generate all the cell types that form the organism. The multipotent stem cells are the only stem cell type found in adults and can be differentiated only towards one linage [32,33,34].

From the point of view of regenerative medicine, there are three types of available stem cells: the stem cells derived from an embryo, the stem cells isolated from adult tissue and the induced pluripotent stem cells [35]. The stem cells isolated from human embryo are difficult to obtain and imply a number of ethical concerns [36], which is why they are extremely rarely used in regenerative studies, especially those involving regeneration of the oral tissue.

The mesenchymal stem cells exosomes have special characteristics, which includes the heterogeneity of contained information depending on the generating type of stem cell. As follows, it is important to analyze the capacity of each cell type to induce angiogenesis and to choose properly the right kind of stem cell for inducing angiogenesis in oral wounds. For instance, plasma from human umbilical cord led to the enhanced re-epithelization and angiogenesis of wounds [37].

The stem cells and their treatment with different growth factors have a significant role in the tissue regeneration [38,39]. The adult human body contains multiple types of stem cells that can become a source of experimentally modulated tissue regeneration. This implies that they can differentiate into a variety of cell types and can emulate with high accuracy the architecture of the original tissue. These are localized in many organs: bone marrow, brain, liver, pancreas, skeletal muscles, skin, and adipose tissue [40].

The regenerative medicine uses various approaches to maintain tissue architecture: from in vivo methods, such as cell-free scaffolds or the use of cell sheet to the ex vivo approaches in which a whole tooth is regenerated from an initial population of stem cells. The use of cell-free scaffolds containing growth factors meant to stimulate the stem cells present at the wound site is an example. For instance, the Guided Tissue Regeneration is a tool used in regenerative medicine, through which a scaffold is placed between the gum and the tooth root in case of injury in order to stimulate the formation of cementum instead of soft tissue [41].

Another example is the use for scaffold free cell sheet which intends to increase the dentistry of implanted MSCs at the wound site and to facilitate intercellular communication [42]. One of the newest methods used in regenerative dentistry is the ex vivo formation of a whole tooth, which is later implanted into the patient [43].

The mesenchymal stromal cells are a diverse type of multipotent cells, which possess high proliferation as well as differentiation potential, with important applications in regenerative medicine. They can be differentiated into adipocytes [44], chondrocytes [45,46], and osteoblasts [47,48].

The stem cells go through two distinct processes: proliferation and differentiation. In order to proliferate, the cells progress through the G1-S-G2-M phases of the cell cycle. For differentiation, the cells remain in the G1-G0 phase. The G0 phase is however a reversible state [49]. There are a number of microRNAs and their target genes that control the balance between proliferation and differentiation [50]. MiR-355 inhibits human mesenchymal stem cells (hMSC) proliferation [51]. The miR-369 stimulates differentiation, by inhibiting proliferation. The miR-369, along with miR-200c and miR-302 are essential stimulators of adult cell reprogramming into a multipotent stem cell [52]. MiR-1, miR-29c, miR-369, miR-371 and miR-499 stimulate proliferation [53]. Depending on the application, the MMSC can be treated with different kinds of molecular mediators and microRNAs [50].

### 2.2. Bone Marrow Mesenchymal Stem Cells (BM-MSC)

Bone marrow mesenchymal stem cells (BM-MSC) represent another source for cell differentiation. The cells in high confluence have a lower proliferation rate and are starting to differentiate. The miR-17, miR-106b, and miR-138 have a lower expression in cells from a high confluence. The miR-663, miR-638, and miR-486 are under-expressed in the cells of 50% confluence and are upregulated in the case of 100% confluence. The TGFβ1, Pigment epithelium-derived factor (PEDF), Thrombospondin 1 (TSP1), insulin like growth factor binding protein 2 (IGFBP2) and metalloproteinase-2 (MMP2) are downregulated, while CD10, CD54, CD106, CD200, and CD282 are upregulated in the case of cells found at 100% confluence [54].

Bone regeneration and the stimulated angiogenesis of a newly formed tissue are important processes for a successful wound healing and regain of function. The BM-MSCs cocultured with Human umbilical vein endothelial cells (HUVEC) lead to successful bone regeneration [55]. In rats, the BM-MSC were cocultured with EC on a scaffold and implanted in a cavity. After 14 days, the tissue was analyzed and it was observed that in the implanted tissue that there is an upregulation of the pro-survival molecule BCL-2, CXCL1, and CXCR2. The CXCL1 is a down-stream effector of BCL-2 activated through the NFkB pathway [56].

### 2.3. Periodontal Ligament Stem Cells (PDLSCs)

Periodontal ligament stem cells (PDLSCs), first described by Gronthos S. et al. [57], are a great source of mesenchymal stem cells. These are originated in the neuronal embryonic layer that has migrated in the oral cavity during embryogenesis. PDLSCs are found in the tooth root and in the alveolar sack [58]. The stem cells from younger donors, especially the ones from deciduous teeth possess a greater regenerative capacity [59]. The dental pulp stem cells (DPSC) can be differentiated in: osteoblasts, adipocytes [60], chondrocytes [61], cementoblasts [59], muscular fibers [62], and mixed neuronal and glial cells [63]. The periodontal stem cells have a higher efficiency of regenerating craniofacial wounds when compared to the more commonly studied BM-MSC [57,64,65]. A commonly used method for enhancing the PDLSCs angiogenic potential is the in vitro treatment with deferoxamine and/or fibroblast growth factor-2. The treatment results in the 1.5–1.8-fold upregulation of VEGF and 5.4–13.1 increase in the level of placental growth factor (PGF). The increased levels are maintained 48h post-treatment [66].

The C-X-C motif chemokine ligand 12 (CXC12) is a factor with essential roles in the homing [67] and motility of BM-MSC [67,68]. However, recent studies have proven its angiogenesis effect [69,70]. The lentiviral overexpression of CXC12 in the PDSC leads to the increased stimulation of the pro-angiogenic factors: VEGF, FGF, PGF, and stem cell factor (SCF) [71]. A recent review has analyses the secretome of dental stem cells and it revealed a series of growth factors many of which are involved in angiogenesis: VEGF, angiopoietin-1 (ANGPT1), angiopoietin-2 (ANGPT2), basic FGF, PDGF, and Insulin-like growth factor-I (IGF-I) [72].

### 2.4. Dental Pulp Stem Cells (DPSC)

Dental pulp stem cells (DPSC), when cocultured with the ECs, stimulate proliferation of the ECs trough the p38 MAPK pathway and induce the overexpression of VEGF-A and MMP-9, while the ANG-1 was downregulated [73]. In a recent study, the DPSCs were transfected with the VEGF and the SDF-1α genes. The transfected cells are more capable of proliferation and inducing the endothelial cells tube formation. The newly formed vascular network has a higher density [74]. 

### 2.5. Gingival Mesenchymal Stem Cells (GMSCs)

In order to reduce the discomfort created by tooth extraction, a procedure needed for the harvesting of periodontal stem cells, some scientist have proposed the use of stem cells derived from gingiva, named gingival mesenchymal stem cells (GMSCs), which have the capacity of rebuilding the cementum, alveolar bone, and the periodontal ligament [75,76].

### 2.6. Induced Pluripotent Stem Cells (iPSCs)

The induced pluripotent stem cells (iPSCs) are a type of cells derived from adult cells [77]. There are two types of experimental strategies: dedifferentiation and reprogramming. Through dedifferentiation, the adult cell becomes a stem cell type from its own lineage, whereas through reprogramming, the cell is reversed to a pluripotent state. The differentiated cells can also pass through a process of transdifferentiation and be directly reprogram to become another cell type [4]. 

The iPSCs injected in an ischemic skeletal muscle led to increased vascular reperfusion, mediated by the enhanced proliferation, motility, and tube formation of endothelial cells due to the elevated levels of VEGF, TGFB1, and angiogenin [78]. In a recent study, the endothelial cells were reprogrammed to iPSCs. In comparison to the mature endothelial cells, the iPSCs from endothelial tissue were less capable of forming mature blood vessels and the density of these blood vessels was lower [79]. Even if the iPSCs have the advantage of being an inexhaustible pool of stem cells, these cells are proposed as a regenerative tool to a far lesser extent, because they can often suffer a number of genetic mutation and can give rise to cancer [80]. 

The angiogenesis induced in vitro may constitute another option for a successful wound healing in cranial wounds. The spheroid MSCs and HUVEC were included in a fibrin scaffold. The HUVECs were stimulated to induce angiogenesis and then the scaffold was implanted. The regenerative process was more successful in this case [81]. 

In Table 1 there is a summary of the characteristics of stem cells available in the oral cavity (Table 1).

## 3. Characteristics of Pro-Angiogenic Exosomes in Regenerative Dentistry

### 3.1. Definition and General Characteristics of Exosomes

Exosomes are extracellular vesicles of 30–100 nm. They belong to a larger category of microvesicles secreted by normal and pathological cells with the property of mirroring cellular content. Initially, exosomes were regarded as a path through which the cells eliminate their waste products. It was later revealed that the exosomes function mainly as mediators of intercellular communication, which maintain the human body homeostasis, as well as several pathological states [82]. 

Exosomes’ cargo is composed of proteins, RNAs [83], and in some cases, even DNA fragments [84]. It distinguishes among the generating cell and it does not constitute random molecules from the cell, instead the cargo is chosen specifically for the delivery of the intended message [85]. In regenerative medicine, this means that the stem cell derived exosomes stimulate proliferation and inhibit the apoptosis of recipient cells, such as epithelial cells, endothelial cells, fibroblasts, and myocytes, thus leading to neovascularization and a balanced tissue repair [86]. 

The exosomes possess specific proteins at their surface. Most of these proteins belong to the following main classes: tetraspanins, annexins and the major histocompatibility complex (I and II) [87]. The surface proteins are necessary for their recognition and internalization by the targeted cell. The exosomes RNA content is composed of mRNAs, microRNA [88,89] and the lesser known nucleic acid species, such as: yRNA [90], rRNA, snRNA, tRNA, snoRNA, piRNA, antisense RNA, scaRNA, circRNA, valut RNA [91], and DNA molecules [92]. 

In the process of exosomes biogenesis several steps are taken. First, the intraluminal vesicles are formed in the multi vesicular body (MVB). The MVB can fuse with a lysosome or the MVB is transported to the plasma membrane [93]. The exosomes are released in the extracellular environment. Then, they can travel through various body fluids and influence the behavior of their targeted cells [94,95]. The exosomes enter into the blood stream or in the lymphatic system and cause changes at distant sites of the body [96,97,98], as it is the case of the metastatic niche preparation, in cancer [99,100,101]. A lesser known travelling medium for exosomes is the saliva, which has major implications for the therapeutic use of exosomes in dental therapies [102,103]. 

When arriving to the targeted cell, the surface molecules recognize the exosomes. The exosomes can directly deliver their content into the cytoplasm of the targeted cell or the exosome can be surrounded by the plasma membrane and be disintegrated in the cytoplasm, where their cargo is released [104,105].

The exosomes are immunomodulators [106]. They repress the T cell activation and are low immunogenetic [107]. The exosomes offer the chance of enhanced regeneration of cells found at a distant site of the body, which helps scientist to overcome the challenges of transfer of information mediated by cell-to-cell contact. 

### 3.2. Cargo of Pro-Angiogenic Exosomes

The stem cells exosomes have special characteristics, which includes the heterogeneity of contained information depending on the generating type of stem cell. As follows, it is important to analyze the capacity of each cell type to induce angiogenesis and to find the right stem cell for inducing angiogenesis in oral wounds [108]. For instance, plasma from the human umbilical cord led to the enhanced re-epithelization and angiogenesis of wounds due to their composition of exosomes rich with miR-21-3p. This microRNA targets sprouty homolog 1 (SPRY1) and phosphatase and tensin homolog (PTEN), two anti-angiogenic factors [37]. 

The stem cells found in the oral cavity secrete exosomes, which have high regenerative capacity. These exosomes transfer information between a stem cell and another cell, leading to the successful architectural replacement of an array of tissue types from the oral cavity, such as the bone or the dental cementum [109]. The regenerative capacity of stem cell exosomes also extends to the regeneration of liver tissue [110] or dopaminergic neurons [111]. This is due to the fact that the MSCs derived exosomes determine the differentiation path of their targeted stem cell [112]. 

The exosomes secreted by stem cells are of high relevance to the regenerative medicine. They stimulate tissue replacement and ensure the proper nurture of the newly formed tissue by stimulating angiogenesis. The pro-angiogenic effects are linked to their capacity to sustain viability and proliferation of endothelial cells [113]. Delta-like 4 (Dll4), a Notch ligand is secreted by the ECs through the exosomes. Its overexpression leads to the inhibition of Notch signaling pathways and filopodia formation. The tube formation assay has shown that the DII4 overexpression leads to enhance vessel formation [114]. MSC-derived multi vesicles (MV) contain surface antigens (CD82, CD9, CD63, CD81, CD109, CD151, CD248, and CD276) [115,116] and fusion proteins (GTPases, annexins, and flotillin) [116].

In order to increase the pro-angiogenic potential of MSC-MVs, there are three different strategies: submitting cells to different environmental stress factors, such as radiation, oxidative stress or hypoxia; transfection with DNA plasmids containing the genes coding for pro-angiogenic factors; and introducing in the MV-producing cell pro-angiogenic factors in a protein form [117]. The maintenance of stem cells in hypoxic conditions has led to an enhanced pro-angiogenic capacity [118], possibly due to the overloading of exosomes with miR-135b, which targets the factor inhibiting HIF-1 (FIH-1) gene, an inhibitor of hypoxia-inducible factor 1 (HIF-1). HIF-1 is a pro-angiogenic factor [119]. 

The DPSC transfected with the HIF-1A gene increased the angiogenesis of EC, through the secretion of exosomes loaded with miR-15/16, miR-31, miR-145, miR-221/222, miR-320a, miR-126, and miR-424 [120].

The exosomes isolated from GMSC and included in a hydrogel/chitosan scaffold increased the in vivo microvessel network and re-epithelization in at the wound site of a rat diabetic model. It causes epithelial growth, collagen remodeling, neuronal growth, and stimulated angiogenesis [121].

The T cells derived exosomes express the thrombospondin-1 and the receptor CD47 on their surface. The EC increase the tube length, while decreasing the number of rings in the newly formed blood vessel. The VEGF signaling pathway activation is dependent on the presence of CD47 [122]. 

BM-MSC secretes exosomes which contain the mRNA for insulin-like growth factor receptor (IGFR). The exosomes, when transferred to the damaged proximal tubular cells caused the regeneration of renal tissue and the induction of IGFR in the renal cells [123]. The IGFR is known to induce angiogenesis of the ECs [124]. The BM-MSC derived exosomes can also bind to the Wnt3a ligand that activates the Wnt/β-catenin pathway, which promotes the network formation by HUVEC cells [125]. 

The growth factors BMP2, BMP6, TGFB1, VEGFA, FGF2, were upregulated in the DPSC cells treated with the differentiation medium -derived exosomes in a 2D culture model, while GDF10, BMP9 and FGF are upregulated in the case of 3D culture model [112]. The stem cells isolated from human exfoliated deciduous teeth secrete exosomes that can reduce the carrageenan-induced edema through the inhibition of MMPs and cathepsin B [126].

**Figure 2:**
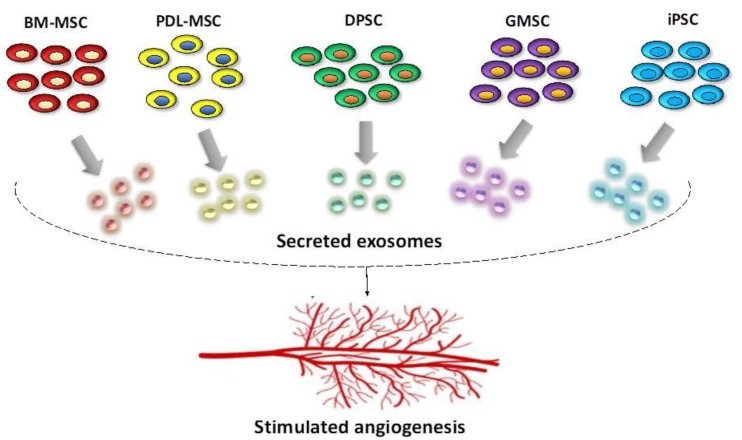
There are multiple stem cells types, which can, through their secreted exosomes, lead to stimulated angiogenesis. In dentistry, there are five main types of stem cells most often evaluated for their general regenerative potential as well as their pro-angiogenic capacity; these are: the bone marrow derived mesenchymal stem cells (BM-MSC), the periodontal ligament stem cells (PDL-MSC), dental pulp stem cell (DPSC), gingival mesenchymal stem cells (GMSC), and induced pluripotent stem cells (iPSC).

The types of stem cells available in the oral cavity and which have the capacity to induce new blood vessel formation are illustrated in Figure 2.

## 4. Key miRNAs with Pro-Angiogenic Effects

The microRNAs are small non-coding RNAs that have strong correlations with many pathologies: cancer [127], cardiovascular diseases [128] and neurodegenerative disease [129].

The MSCs secrete exosomes with strong pro-angiogenic capacity through their miRNA cargo and its targets in the recipient cell. For instance, miR-21 activates the AKT and ERK pathway thus leading to the VEGF overproduction. MiR-1246 is involved in angiogenesis by inhibiting the expression of PML, an inhibitor of the SMAD 1/5/8 pathway. MiR-23a family is strongly associated with increased angiogenesis, being overexpressed in the lung and heart tissue [56]. 

The microRNAs direct the EndoMT process during neovascularization [130]. In HUVEC cells, the treatment with TGF-β induces miR-21 overexpression and EndoMT stimulation. Phosphatase and tensin homolog (PTEN) expression is downregulated as a consequence of miR-21 upregulation [131]. This non-coding RNA was found in the BM-MSC exosomes [132] and may have a protective role against periodontal inflammation [133]. 

The BM-MSC treated with miR-378 mimic developed a higher density of capillary tube network, in the Matrigel angiogenesis assay. This was mediated also by an induced overexpression of VEGF and ANG [134].

The miR-130a is a strong positive regulator of angiogenesis. It targets the anti-angiogenic factors: growth arrest-specific homeobox (GAX) and homeobox A5 (HOXA5) [135]. The loading of miR-130a-3p in the exosomes of MSC results in the stimulation of HUVEC-mediated neovasculature [56]. 

The miR-126 is another miRNA specifically overexpressed in endothelial cells which exerts its activity by targeting the inhibitor of angiogenesis Spred-1 [136]. This miRNA was found in the exosomes from endothelial progenitor cells [137]. MiR-125b is another pro-angiogenic miRNA which targets p53 and promotes EndoMT [138].

The let-7f and miR-27b are overexpressed in EC and stimulate angiogenesis [139]. The Dentin matrix protein 1 (DMP1), an angiogenic inhibitor [140] is a target of let-7f in dental pulp cells [141].

MiR-214 was found in the exosomes from the endothelial cells and it induces the viability and migration of ECs during new blood vessel formation [142]. 

## 5. Key miRNAs with Anti-Angiogenic Effects

The miR-15/107 group is composed of: miR-103 1/2, miR-107, miR-646, miR-503, miR-424, miR-15b, miR-497, miR-15a, miR-195, miR-16-1/-2 [143]. They have extensive roles in angiogenesis. The miR-503 impairs the EC hypoxia-induced proliferation and migration; at the same time causing cell cycle arrest. In the case of endothelial progenitor cells, the reported targets of miR-503 are: CDC25A, CCND1/2, CCNE1 [144], and apelin [145].

The transfection of miR-16 in ECs, causes VEGF, VEGFR2, and FGFR1 inhibition and the loss of cell viability [146]. The miR-16 was found in the extracellular vesicles from MSC [147]. The miR-16-1 was found to be downregulated in DPSC [148].

The role of wound healing impairment of miR-15b has been proven also in the case of diabetes [149]. The inhibition of miR-15 has a positive effect over neovascularization. This microRNA is downregulated in the exosomes from BM-MSC, compared to the generating BM-MSC [150]. During aortic development miR-15 and miR-29 have increased expression, which results in the downregulation of elastin gene (ELN) [151]. The elasticity of each blood vessel is orchestrated by the vascular smooth muscle, a tissue type with high elastin content [152]. The miR-29 was found to be downregulated in DPSC in comparison with BM-MSC [148]. In ischemic tissue of rats, it was proven that miR-103 targets the VEGF and that its inhibition leads to increased tube length and migration of the EC [153]. This microRNA has a higher expression level in response to hypoxia, along with miR-23, -24, -26, -27, -107, -181, -210, and -213 [154]. 

The miR-424 is another member of miR-16 family with inhibitory roles in angiogenesis, by causing EC impairment of vascular sprouting, migration, and tube formation. Its expression, as well as the expression of miR-503 is regulated by PPARγ [155]. In senile haemangioma, the mir-424 inhibition causes a greater proliferation rate of human dermal microvascular ECs [156]. The induced overexpression of miR-424 in ECs, causes the downregulation of VEGF, VEGFR-2, and FGFR-1, while it upregulates miR-16-1/2 [146]. In the human dental pulp cells (hDPCs), it was proven that the inhibition of miR-424 causes elevated tube formation and endothelial differentiation. This study also showed that the vWF, KDR, and VEGF are putative targets of this non coding RNA (ncRNA) [157]. The dental pulp stem cell (DPSC) induced to differentiate into endothelial cells by hindering the expression of miR-424 [157]. 

The miR-200b is another negative regulator of angiogenesis. The expression level of this ncRNA decreases in hypoxic conditions [158]. During wound healing the expression of miR-200b is downregulated, while GATA2 and VEGFR2 are upregulated [159].

The miR-200 family blocks tumor angiogenesis and migration [160]. In the EC, it was proven that miR-200b targets the VEGF, FLT-1, and KDR thus impairing tube formation [161]. This miRNA also inhibits EndoMT by targeting the Zinc Finger E-Box Binding Homeobox 1 (ZEB1) and the Zinc Finger E-Box Binding Homeobox 2 (ZEB2) genes [162]. 

The silencing of this ncRNA stimulates a better wound healing [159]. The miR-200 family, as well as the miR-205 are important regulators of cell migration by binding to the ZEB1 and SIP1 mRNAs [163].

The miR15b and miR200b target the VEGF, VEGFR, and ANG-1 thus inhibiting angiogenesis. In diabetic mice, these miRNAs are overexpressed at the wound site [164]. The reduced angiogenic capacity of diabetic mice can be restored with the help of an ingenious scaffold design. The collagen coated scaffold proved an equal capacity of new blood formation in the case of both control and diabetic mice [165].

A list of studies focusing on miRNAs secreted by stem cells is found in Table 2.

**Table 1:** Types of stem cells used in regenerative dentistry, their source, differentiation potential and the alteration they cause inside endothelial cells, in the angiogenesis related genes.

Type of Stem Cells	Source	Differentiation Potential	Alteration of Angiogenesis Related Genes	Ref.
BM-MSC	Non-hematopoietic components of the BM	osteoblasts, adipocytes, chondrocytes, smooth muscle, sarcomeric muscle, endothelial, neural and hepatocytic lineages	Upregulation of BCL-2, CXCL1 and CXCR2	[166,167,168]
PDLSC	Mature periodontal ligaments	adipocytes and osteoblasts, cementoblasts, fibroblasts, adipocytes, and chondroblasts,	Upregulation of VEGF and FGF-2	[59,66]
DPSC	Human exfoliated deciduous teeth, apical papilla, periodontal ligament and dental follicle tissue.	osteoblasts, rare pancreatic islands, endothelial and smooth muscle cells	Upregulation of BMP2, BMP6, TGFB1, VEGFA, FGF2	[112,169,170]
GMSC	Gingival connective tissue as gingival mesenchymal stem/progenitor cells	osteoblasts, adipocytes, chondrocytes, endothelial and neural cells	N/A	[75,171]
iPSC	Different types of adult cells	fibroblast, adipocytes, cardiomyocytes, pancreatic cells, neural cells	Upregulation of VEGF, TGFB1 and ANG	[78,172]

BM—bone marrow, BM-MSC—mesenchymal stem cell from bone marrow, PDLSC—Periodontal ligament stem cells, DPSC—dental pulp stem cells, GMSC—gingival mesenchymal stem cells, and iPSC—induced pluripotent stem cells, N/A—not applicable.

## 6. Current Challenges

The modulation of angiogenesis and the crosstalk between endothelial cells and stem cells is a therapeutic approach increasingly used in regenerative dentistry. Moreover, the translation of this research is very promising, considering that there are now two clinical trials using a similar strategy in cardiovascular disease (NCT00314366, NCT01468064). 

The most commonly used approach is the coculture of the MSCs with the endothelial cells or a scaffold containing both. This is the most studied method in the current preclinical studies. However, the stem cells kept in in vitro conditions need to maintain viability and proliferate in an enough number for a successful transplantation. 

The bone marrow mesenchymal stem cells are the best option for this use, but they have a limited capacity to induce angiogenesis in oral wounds and during harvesting, it implies major discomfort. The best option of mesenchymal stem cells is the orally available stem cells that are easier to harvested, however this approach lacks procedure standardization. As follows, more research should be developed in this direction. 

Moreover, the cell therapy approach leads many times to an immune reaction and it poses the danger of malignant transformation. Part of the solution to this problem is the choice of low tumorigenic and immunogenic stem cells and avoiding the well-known cancer-prone cells, such as induced pluripotent stem cells. 

Another option is the cell-free therapy in which the mesenchymal stem cells from the oral tissue are cultivated and their exosomes are harvested. The stem cells can be genetically modified to stimulate enhanced levels of pro-angiogenic factors or the isolated exosomes can be loaded with additional pro-angiogenic cargo. Evidence supports the superiority of this approach over that of the stem cell therapy. However, there are some drawbacks regarding the failure to generate a large enough quantity of exosomes, sample impurity and the inability to expand the number of exosomes without the use of cells. Moreover, if in the receiving organism there are cancerous cells, the MSCs exosomes stimulate cancer progression.

The microRNAs, as master regulators of gene expression can constitute the main focus of pro-angiogenic exosome therapy. The MSCs exosomes are enriched in microRNAs targeting anti-angiogenic genes. Still, some MSCs exosomes may be less efficient in promoting new blood vessel formation at the wound site. This ability can be influenced by the local microenvironment, which is why it is extremely important to analyze the therapeutic exosomes from the point of view of pro-angiogenic miRNAs, mRNAs, and proteins. 

**Figure 3:**
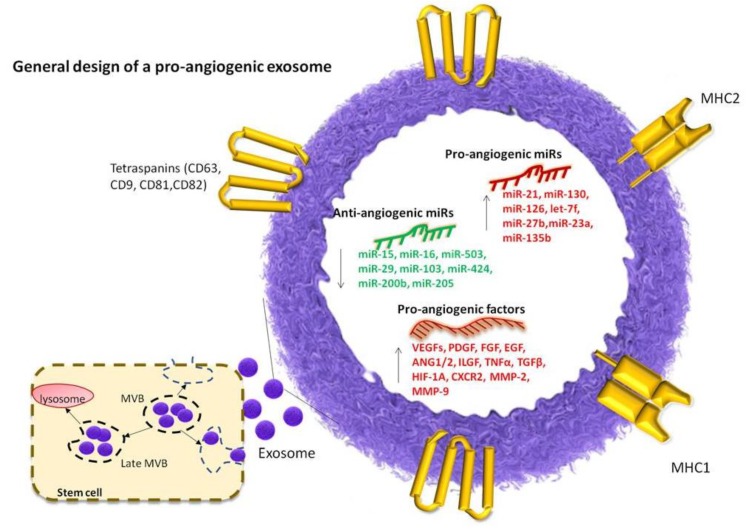
The exosomes are primarily located in the multivesicular body (MVB) of the generating cell. The MVB can become late MVB. At this stage the late MVB can fuse with a lysosome and degrade its content, or it can release the exosomes in the extracellular environment. The exosome has surface proteins which help it to be recognized by the targeted cell. Among the surface proteins there are: tetraspanins (CD63, CD9, CD81, CD82), MHC I, MHC II annexins, lipid rafts, etc. At its interior, a pro-angiogenic exosome will carry proangiogenic miRNAs- such as miR-21, miR-23, miR-1246, miR-378, miR-16 family, miR-142, miR-196a, miR-17, miR-2861, miR-210, miR-20a, miR-29a, miR-10a/b, miR-126, miR-19a/b, miR-125a, miR-15, miR-16, miR-17, miR-31, miR-126, miR-145, miR-221, miR-222, miR-320a, miR-424—and proangiogenic factors in the form of mRNAs or proteins—VEGF, FGF, ANG1/2, ILGF, HIF-1A, MPP9, CXCR2 etc.

Our proposal of cell-free, MSCs derived exosomes loaded with pro-angiogenic miRNAs, constitutes a future option for a successful tissue nutrient supply and regeneration at the wound site. Still, there are a number of issues that need to be addressed and standardized. We illustrated our therapeutic strategy and the general design of a pro-angiogenic exosome in Figure 3. 

## 7. Conclusions

The exosomes derived from the stem cells available in the oral cavity and directed towards the local endothelial cells constitute promising therapeutic options for the stimulated angiogenesis in regenerative dentistry. The exosome secreting stem cells that are utilized for this kind of approach are: bone marrow stem cells, dental pulp stem cells, periodontal ligament stem cells, gingival mesenchymal stem cells, and induced pluripotent stem cells. These cells can be cultivated in stress-inducing conditions, such as hypoxia, or they can be transfected with pro-angiogenic genes, will result in the secretion of more pro-angiogenic exosomes. The exosomes themselves can be isolated and manipulated ex vivo to contain a more pro-angiogenic cargo. The cargo of these exosomes should contain molecular mediators such as: VEGFs, PDGF, FGF, EGF, ANG1/2, ILGF, TNFα, TGFβ, HIF-1A, CXCR2, MMP2/9; as well as pro-angiogenic microRNAs, such as miR21, miR-23, miR-1246, miR-378, miR-16 family, miR-142, miR-196a, miR-17, miR-2861, miR-210, miR-20a, miR-29a, miR-10a/b, miR-126, miR-19a/b, miR-125a, miR-31, miR-145, miR-221/222, miR-126, miR-320a, miR-424. Because the stem cells and the EC have a strong interconnection, maintained especially by the exosomes, the therapeutic stimulation of angiogenesis in regenerative dentistry should consider the synchronous treatment of all the involved factors.

## Figures and Tables

**Table 2:** Different types of pro-angiogenic exosome recreating cells, their cargo/targeted mRNAs, microRNAs or proteins, the resulted upregulation or downregulation and the biological changes made in the targeted cells (in this case the endothelial cells -ECs).

Type of Secreting Cell	Up/Down Level	Cargo/Targeted Molecules	Biological Changes of the Targeted Cell (EC)	Ref.
DP- MSC. Lentiviral transfected with HIF-1A	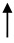	miR-15/16, miR-17, miR-31, miR-221/222, miR-320a, miR-424 miR-126, miR-145	Stimulation of angiogenesis	[120]
MSC	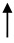	*STAT3, C-MYC, CYCLIN A1, CYCLIN D2*	Induced vascular tube formation, Activate several intracellular signalling pathways	[173]
	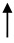	miR-10a/b, miR-21, miR-19a/b miR-126,	Promote angiogenesis	[174]
Pla-MSC and BM-MSC	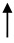 (BMMSC)	*VEGF, HGF, IGFBP2, IGFBP6*	Increased endothelial tube formation and migration	[175]
	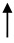 (Pla-MSC)	*HGF, IGFBP2, IGFBP3, IGFBP6*		
iMSC	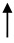	*VEGF, TGFB1, ANG*	Promotes EC migration, proliferation Increased tube formation	[78]
AD-MSC	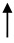	miR-125a	EC pro-angiogenic activity	[176]
	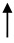	*ANGPT1, FLK1, HIF-1A VEGF*	Increased tube formation	[177]
	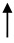 (Nrf2+ ADSC)	SMP30, VEGF, VEGFR2 phosphorylation,	Inhibited ROS and inflammatory cytokine expression Inhibited EC senescence	[178]
HucMSC	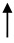	*PCNA, CYCLIN D3, N-CADHERIN*	Proliferation and Migration of EC Improved the tube-formation ability of EC	[179]
DPC	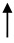	*FGF-2, VEGF-A, KDR, MMP-9*	Promoted increased tube formation EC proliferation	[67]
GMSC	N/A	N/A	A higher number of newly formed microvesicles	[121]
MSC	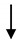	miR-424, miR-30c, miR-30b, and let-7f	Promote angiogenesis	[174]
AD-MSC	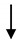	*DLL4*	EC pro-angiogenic activity	[176]
AD-MSC	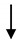	*VASH1*	Increased tube formation	[177]
AD-MSC, Nrf2+AD-MSC	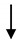 (ADSC)	SMP30, VEGF	Inhibited ROS and inflammatory cytokine expression Inhibited EC senescence	[178]
HucMSC	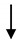	*E-CADHERIN*	Proliferation and Migration of EC Improved the tube-formation ability of EC	[179]

MSC—mesenchymal stem cells, DP-MSC—mesenchymal stem cells from dental pulp, PlaMSC—placenta-derived mesenchymal stem cells, BM-MSC—mesenchymal stem cells from bone marrow, iMSC—induced mesenchymal stem cells, ad-MSC—adipose tissue mesenchymal stem cells, HucMSC—human umbilical cord mesenchymal stem cell, AD-MSC—adipose tissue mesenchymal stem cells, DPC—dental pulp cells, GMSC—gingival mesenchymal stem cells, N/A—not applicable.
